# Modelling idiopathic intracranial hypertension in rats: contributions of high fat diet and testosterone to intracranial pressure and cerebrospinal fluid production

**DOI:** 10.1186/s12987-023-00436-1

**Published:** 2023-06-16

**Authors:** Jonathan H. Wardman, Mette N. Jensen, Søren N. Andreassen, Bjarne Styrishave, Jens E. Wilhjelm, Alexandra J. Sinclair, Nanna MacAulay

**Affiliations:** 1 Department of Neuroscience, Faculty of Health and Medical Sciences, University of Copenhagen, Blegdamsvej 3, 2200 Copenhagen N, Denmark; 2 Department of Pharmacy, University of Copenhagen, Copenhagen, Denmark; 3 Department of Health Technology, The Technical University of Denmark, Kgs. Lyngby, Denmark; 4 Institute of Metabolism and Systems Research, University of Birmingham, Birmingham, UK

**Keywords:** Idiopathic intracranial hypertension, Choroid plexus, IIH, Cerebrospinal fluid, NKCC1, Testosterone, Androgens, Intracranial pressure

## Abstract

**Background:**

Idiopathic intracranial hypertension (IIH) is a condition characterized by increased intracranial pressure (ICP), impaired vision, and headache. Most cases of IIH occur in obese women of childbearing age, though age, BMI, and female sex do not encompass all aspects of IIH pathophysiology. Systemic metabolic dysregulation has been identified in IIH with a profile of androgen excess. However, the mechanistic coupling between obesity/hormonal perturbations and cerebrospinal fluid dynamics remains unresolved**.**

**Methods:**

Female Wistar rats were either fed a high fat diet (HFD) for 21 weeks or exposed to adjuvant testosterone treatment for 28 days to recapitulate IIH causal drivers. Cerebrospinal fluid (CSF) and blood testosterone levels were determined with mass spectrometry, ICP and CSF dynamics with in vivo experimentation, and the choroid plexus function revealed with transcriptomics and ex vivo isotope-based flux assays.

**Results:**

HFD-fed rats presented with increased ICP (65%), which was accompanied by increased CSF outflow resistance (50%) without altered CSF secretion rate or choroid plexus gene expression. Chronic adjuvant testosterone treatment of lean rats caused elevated ICP (55%) and CSF secretion rate (85%), in association with increased activity of the choroid plexus Na^+^,K^+^,2Cl^−^ cotransporter, NKCC1.

**Conclusions:**

HFD-induced ICP elevation in experimental rats occurred with decreased CSF drainage capacity. Adjuvant testosterone, mimicking the androgen excess observed in female IIH patients, elevated the CSF secretion rate and thus ICP. Obesity-induced androgen dysregulation may thus contribute to the disease mechanism of IIH.

**Supplementary Information:**

The online version contains supplementary material available at 10.1186/s12987-023-00436-1.

## Introduction

Idiopathic intracranial hypertension (IIH) is characterized by an unexplained increase in intracranial pressure (ICP) resulting in life-altering symptoms. These include severe, persistent headache and optic nerve swelling (papilloedema) that can lead to permanent visual loss and cognitive impairment [1–6]. Obese women of childbearing age make up approximately 90% of all IIH cases, though IIH can rarely afflict males, diverse ages, and body-types [5–8]. Increased global rates of obesity have led to increased IIH incidence (by greater than 350% in the last decade) [7, 9–11]. Visual impairments have improved due to increased awareness and earlier intervention [11–13], but severe headache and cognitive dysfunction continue to threaten those suffering from IIH, which can lead to long-term disability [3, 14, 15]. Pharmacological interventions for IIH are currently limited to unlicensed used of diuretic drugs such as acetazolamide and topiramate [6], which are comprised by side-effects and low adherence [2]. Severe IIH, with rapidly progressive visual loss, remains treatable only via surgical procedures such as ventriculo-peritoneal shunting, which is associated with marked complications [2]. Weight loss via dietary restriction or bariatric surgery has proven effective for reducing ICP and inducing remission in IIH [16, 17]. This apparent link between weight loss and symptom alleviation indicates a connection, currently unresolved, between obesity and the etiology of IIH [17].

The elevated ICP in IIH has been attributed to multiple factors, including increased venous pressure [18, 19], aspects of CSF dynamics such as elevated brain fluid content [20–22] due to inefficient CSF clearance [23–26], increased CSF production rate [23, 27], or venous sinus stenosis [28, 29], as well as reduced compliance of brain tissue and vasculature due to fibrogenesis and deterioration of the basement membrane, among others[30–34]. Some of these suggested etiologies have, however, been contested [35–37]. Despite a clear link between female sex, obesity, and age and the symptoms of IIH, IIH only affects up to 15.2/100,000 patients fitting these criteria [7], and IIH is not endemic in the obese female population, suggesting the presence of other factors leading to emergent IIH. A range of factors of putative importance for disease etiology has been detected in IIH patients [38–40]. Most notably, analysis of IIH steroid hormone profiles revealed a unique phenotype of serum and CSF androgen excess, distinct from that driven by obesity alone [39, 41]. The interplay between adiposity and androgen excess, both features of systemic metabolic dysregulation, and ICP elevation is not understood.

Pathological alterations in ICP can be attributed to disturbed CSF dynamics; the balance between CSF production, circulation, and drainage [42, 43]. CSF produced primarily by the choroid plexus disperses into brain compartments including the ventricles and the subarachnoid space prior to exiting via lymphatic and/or perineural pathways or the arachnoid granulations [42, 44]. Hence, increased CSF production or reduced CSF drainage may disturb normal brain fluid content or distribution and thus modulate the ICP [45–48]. Recent studies have revealed the importance for CSF secretion of various choroid plexus transport mechanisms, the Na^+^/K^+^-ATPase and the Na^+^/K^+^/2Cl^−^ cotransporter (NKCC1)[42, 49, 50], the activity of which could have a role in ICP pathologies, including IIH.

Obesity and androgen profiles are not readily manipulated in humans in order to determine their individual impact on ICP, brain fluid dynamics, and choroid plexus function. We therefore aimed to resolve the role of obesity and androgen excess on CSF dynamics in an animal model. Previous studies have focused on obesity as the major factor to recapitulate in an animal model of IIH, using obesogenic diets [51–53]. Similarly, we employed a high fat diet (HFD)-fed rat model mimicking aspects of IIH [51, 53] and a complementary rat model of elevated testosterone for determination of the ensuing brain fluid dynamics to elucidate their potential contribution to the ICP elevation that defines IIH.

## Methods

### Experimental animals

Animal handling and experiments were performed according to European guidelines and complied with all ethical regulations. It was approved by the Danish Animal Experiments Inspectorate with permission no. 2018-15-0201-01515. Female Wistar rats (Janvier Labs), aged 6 weeks, were divided into two groups of equal starting weight (HFD = 171 ± 10 g, n = 17, control = 173 ± 9, n = 14, p = 0.98) and were fed either a high fat diet (HFD, 60% Kcal from fat, Research Diets: D12492i) or a nutrient source-matched control diet (10% Kcal from fat, Research Diets: D12450ji) for 21 weeks. Diet and water were provided ad libitum and the rat weight recorded on a weekly basis. Experimental animals were chosen based upon weight, selecting control diet-fed rats weighing below a 305 g threshold and HFD-fed rats above a 335 g threshold, providing an 18% average weight difference between groups, similarly to weight differences and thresholds obtained in a previous study [53]. For testosterone experiments, female Wistar rats (Janvier Labs) were used at nine weeks of age, with either 2 weekly subcutaneous injections starting 4 weeks prior with 1 mg testosterone propionate in 100 µl sesame oil (86,541, Sigma-Aldrich) [54] or 100 µl sesame oil (S3547, Sigma-Aldrich, vehicle control). Testosterone administration was calculated to obtain supraphysiological testosterone levels comparable to those observed in female IIH patients [39, 54].

### Solutions

The majority of the experiments were conducted in CO_2_/HCO_3_^−^-buffered artificial cerebrospinal fluid (HCO_3_^−^-aCSF; (in mM) 120 NaCl, 2.5 KCl, 2.5 CaCl_2_, 1.3 MgSO_4_, 1 NaH_2_PO_4_, 10 glucose, 25 NaHCO_3_, equilibrated with 95% O_2_/5% CO_2_ to obtain a pH of 7.4). In experiments where the solution could not be equilibrated with 95% O_2_/5% CO_2,_ as in isotope influx and efflux experiments, aCSF was instead buffered by HEPES (HEPES-aCSF; (in mM) 120 NaCl, 2.5 KCl, 2.5 CaCl_2_, 1.3 MgSO_4_, 1 NaH_2_PO_4_, 10 glucose, 17 Na-HEPES, adjusted to pH 7.4 with NaOH).

### Anesthesia and ventilation

Anesthesia was implemented via intraperitoneal (i.p.) injection of 6 mg/ml xylazine + 60 mg/ml ketamine (ScanVet) in sterile water (0.17 ml/100 g bodyweight, pre-heated to 37 °C). Animals were re-administered half ketamine dose as required to sustain anesthesia. One rat was excluded because it was unresponsive to initial anesthesia administration. The body temperature was maintained at 37 °C by a homeothermic monitoring system with heat pad (Harvard Apparatus). Mechanical ventilation was employed for all anesthetic protocols lasting more than 30 min, to ensure stable respiratory partial pressure of carbon dioxide (pCO_2_) and oxygen (pO_2_) and arterial oxygen saturation and thus stable plasma pH and electrolyte content. Surgical tracheotomy was carried out for mechanical ventilation, which was controlled by the VentElite system (Harvard Apparatus) by 0.9 l/min humidified air mixed with 0.1 l/min oxygen adjusted with approximately 2.6 ml per breath, 80 breath/min, a Positive End-Expiratory Pressure (PEEP) at 2 cm, and 10% sight for a ~ 350 g rat. Ventilation settings were optimized for each animal using a capnograph (Type 340, Harvard Apparatus) and a pulse oximeter (MouseOx® Plus, Starr Life Sciences) after system calibration with respiratory pCO_2_ (4.5–5.0 kPa) and pO_2_ (13.3–17.3 kPa) and arterial oxygen saturation (98.8–99.4%) (ABL90, Radiometer).

### ICP measurements

Anesthetized and ventilated rats, placed in a stereotactic frame, had the skull exposed, and a 3.6 mm diameter cranial window drilled with care not to damage the dura. The epidural probe (PlasticsOne, C313G) was secured with dental resin cement (Panavia SA Cement, Kuraray Noritake Dental Inc.) above the dura and the ICP probe was filled with HEPES-aCSF before connection to a pressure transducer APT300 connected to a transducer amplifier module TAM-A (Hugo Sachs Elektronik) followed by a multifunction data acquisition module DT9836-12-2-BNC (Data Translation). To ensure the presence of a continuous fluid column between the dura and the epidural probe, approximately 5 µl HEPES-aCSF was injected through the epidural probe. The ICP signal was recorded at a 1 kHz sampling rate using BDAS Basic Data Acquisition Software (Hugo Sachs Elektronik). Jugular compression was applied to confirm proper ICP recording. After at least 20 min of ICP recording, the resistance to CSF drainage assay was performed by infusing aCSF into the lateral ventricle alzet for intervals of 10 min at 5, 10, 15, and 20 µl/min, with a one minute pause between each rate increase. Resistance to drainage (*R*_*out*_) was calculated using the following equation (derived from [55]).

$${R}_{out}=\frac{{ICP}_{f}-{ICP}_{i}}{I}$$

where *ICP*_*f*_ = stable ICP after 10 min of infusion at a given rate in mmHg, *ICP*_*i*_ = initial, stable baseline ICP in mmHg, and *I* = infusion rate in µl/min. *R*_*out*_ for each rate of infusion was calculated and averaged across all infusion rates for each rat to determine resistance to CSF drainage.

### ICP waveform analysis

The raw ICP data stored as semicolon-separated values in normal text files by the BDAS software were read into MATLAB (MathWorks), and possible spikes and non-physiological data points removed. This was followed by low-pass filtering (anti-aliasing) and downsampling to 100 Hz. These signals were of length about 300 s to 900 s and contained approximately 1.3 Hz frequency components from the artificial ventilation, the heart rate as well as other minor interferences. Since the heart rate was in the region 2.5 to 5 Hz, the signal was spectrally bandpass filtered between 1.6 Hz and 5.5 Hz with a Tukey window. The instantaneous peak-to-peak amplitude was finally extracted from the filtered time signals and employed to obtain the mean wave amplitude.

### Brain water quantification

For brain water determination, rats were decapitated under anesthesia. The brain was rapidly dissected into a pre-weighed porcelain evaporating beaker (Witeg) and weighed within 1 min after brain isolation**.** The brain was then homogenized with a spatula (to increase surface area) and left at 100 °C for approximately 90 h to dry. After drying, the brain was weighed again and the difference in the two measurements corresponded to the brain water (in g) and was employed to obtain the water percentage.

### Ventriculo-cisternal perfusion

Rats were anesthetized, ventilated, and an infusion cannula (Brain infusion kit 2, Alzet) was stereotactically placed in the right lateral ventricle. A 0.5 mm (diameter) burr hole was drilled (1.3 mm posterior, 1.8 mm lateral to bregma), and a 4 mm (length) brain infusion cannula (Brain infusion kit2, Alzet) was glued in place on the cranium with the cannula placed into the lateral ventricle, through which a pre-heated (37 °C, SF-28, Warner Instruments) HCO_3_^−^-aCSF containing 1 mg/ml TRITC-dextran (tetramethylrhodamine isothiocyanate-dextran, MW = 150000; T1287, Sigma) was infused at 9 µl/min. CSF was sampled from cisterna magna at 5 min intervals with a glass capillary (30–0067, Harvard Apparatus pulled by a Brown Micropipette puller, Model P-97, Sutter Instruments) placed at a 5° angle (7.5 mm distal to the occipital bone and 1.5 mm lateral to the muscle-midline). The cisterna magna puncture and continuous fluid sampling prevents elevation of ICP during the procedure. The fluorescent content of CSF outflow was measured in triplicate on a microplate photometer (545 nm, SyneryTM Neo2 Multi-mode Microplate Reader; BioTek Instruments), and the CSF secretion rate was calculated from the equation [56]:

$$Vp=ri\times \frac{Ci-Co}{Co}$$

where *V*_*p*_ = CSF secretion rate (µl/min), *r*_*i*_ = infusion rate (µl/min), *C*_*i*_ = fluorescence of inflow solution, *C*_*o*_ = fluorescence of outflow solution.

### Live imaging of CSF movement

Rats were anesthetized, placed in a stereotactic frame and through a burr hole in the lateral ventricle (same coordinates as for ICP and ventriculo-cisternal perfusion) a Hamilton syringe (RN 0.40, G27, a20, Agntho’s) was placed (4 mm deep) with 15 μl HCO_3_^−^-aCSF with 10 μM carboxylate dye (MW = 1,091, IRDye 800 CW, P/N 929-08972, LI-COR Biosciences). For the acute testosterone treatment, an additional Hamilton syringe containing 15 μl HCO_3_^−^-aCSF with 2.5 μM testosterone (or 0.0025% DMSO as vehicle control) was injected into the ventricle five minutes prior the dye injection. The rat was swiftly placed in a Pearl Trilogy Small Animal Imaging System (LI-COR Biosciences) and within 1 min after ventricular dye injection, images were obtained at 30 s intervals (800 nm channel, 85 μm resolution, for 5 min). A white field image was acquired at the termination of each experiment, after which the rat was sacrificed. The isolated brain was then bisected to expose the ventricles to record a final micrograph ensuring proper targeting of the ventricular compartment. Images were analyzed in a blinded fashion using LI-COR Image Studio 5.2 (LI-COR Biosciences) and data presented as fluorescence intensity in a region of interest placed in line with lambda, normalized to the signal obtained in the first image, in arbitrary units (a.u.). This assay has been verified against the gold standard ventriculo-cisternal perfusion assay in a several publications, where inclusion of inhibitors yielded similar results with the two assays [46, 49, 57].

### Androgen quantification in CSF and plasma

Rats were anesthetized, placed in a stereotactic frame and CSF was extracted through a cisterna magna puncture and immediately centrifuged to pellet cell debris (2000 ×*g*, 10 min, 4 °C) prior to storage of the ~ 100 µl supernatant at − 80 °C in sealed microcentrifuge tubes. Immediately after euthanization of each rat, a blood sample was collected into heparin-coated Eppendorf tubes and centrifuged (7500 ×*g*, 5 min, 4 °C). The ~ 400 µl plasma was transferred to new vials and stored at − 80 °C until analysis. The steroid extraction was performed as earlier described for plasma [58] with a further modification of the CSF analysis to encompass a different size of solid-phase extraction columns (100 mg Bond elute C_18_ solid-phase extraction cartridges; 1 ml; Agilent) and therefore corresponding volume changes for conditioning (1 ml MeOH followed by 2 × 1 ml dH_2_O), washing (2 × 1 ml dH_2_O followed by 1 ml H_2_O:MeOH (3:1)) and elution (1 ml H_2_O:MeOH (1:4)). The liquid chromatography online clean-up, chromatographic separation of androgens, and mass spectrometry data analysis were done as earlier described [58].

### RNA sequencing

Choroid plexus (lateral and 4th) was isolated and stored in RNAlater^®^ (Sigma) at − 80 °C prior to RNA extraction and library preparation with NEB Next^®^ Ultra^™^ RNA Library Prep Kit (NEB) by Novogene. RNA sequencing (paired-end 150 bp, with 12 Gb output) was performed on an Illumina NovaSeq 6000 (Illumina). All program parameter settings for library building and mapping, together with all scripts for the gene annotation and analysis are available at https://github.com/Sorennorge/-MacAulayLab-RNAseq3-Wistar. Raw data are available at the National Center for Biotechnology Information (NCBI) Gene Expression Omnibus (GEO) database (GSE223582). The sequencing data of 150 base paired-end reads were mapped to reference genome (Rattus norvegicus Rnor_6.0 v.104) using Spliced Transcripts Alignment to a Reference (STAR) RNA-seq aligner (v. 2.7.9a) [59]. The mapped alignment by STAR was both converted to raw counts from STAR GeneCounts and normalized to TPM with RSEM (RNA-Seq by Expectation Maximization v. 1.3.3) [60]. The raw counts from STAR GeneCount were used for differential expression analysis using R library and program DEseq2 [61]. Differentially expressed genes were determined based on standard procedure of DEseq2 analysis with false discovery rate (FDR, Benjamini–Hochberg method) [62] of less than 0.05 [63]. The Volcano plot was created using R library ggplot2 [64], the subplot for the pie chart was created using python library matplotlib, and the subplot for Heatmap was generated using R library pheatmap [65]. The Gene Ontology (GO) enrichment analysis was generated utilizing the Panther database [66] with the gene symbols from the differentially expressed genes from DEseq2 to classify the protein class of each gene and the pie chart of the GO enrichment analysis created using python library matplotlib. The network analysis was generated from differentially expressed genes from DEseq2 using the gene symbols as protein database query from String-database (https://string-db.org/) [67] and only including connections with a string confidence score above 0.7 as a plugin for Cytoscape (v. 3.9.1) [68].

### ^86^Rb^+^ influx and efflux

Lateral choroid plexus was isolated from control and testosterone-treated rat brains and placed in 37 °C HEPES-aCSF for a 5–10 min recovery period followed by 2 min (influx) or 8 min (efflux, with inclusion of bumetanide (20 µM) or vehicle) of incubation in an isotope solution containing rubidium (^86^Rb^+^) (1 µCi/ml, 022-105721-00321-0001, POLATOM) and ^3^H-mannitol (4 µCi/ml, NET101, Perkin Elmer). ^86^Rb^+^ acts as a K^+^ congener, and can be transported by the Na^+^, K^+^, 2Cl^−^ cotransporter, NKCC1, and the Na^+^/K^+^-ATPase, amongst others, in place of K^+^, whereas ^3^H-mannitol remains outside, serving as an extracellular marker [69]. The ^86^Rb^+^ transport rate is independent of whether the aCSF is buffered by HCO_3_^−^ or HEPES [47] and the experiments thus conducted in HEPES-buffered aCSF to avoid the required equilibration of the HCO_3_^−^-buffered aCSF, which may induce spraying of isotopes. For efflux assays, choroid plexuses were, in a paired fashion, randomly assigned to either control or bumetanide group (20 µM, Sigma, B3023, stock solution prepared in DMSO (Sigma D8418) prior to dilution in HEPES-aCSF to a final DMSO concentration of 0.1% as also employed as vehicle). For influx assays, choroid plexuses were, in a paired fashion, randomly assigned to either control or ouabain group (2 mM, Sigma, O3125, dissolved directly into HEPES-aCSF on the day of experiment). The acute exposure to testosterone consisted of a one hour preincubation of the choroid plexus in 37 °C HEPES-aCSF containing 100 nM testosterone (dissolved in ethanol prior to dilution in HEPES-aCSF to a final vehicle concentration of 0.04% ethanol, which was employed in the control group) prior to initiation of the flux assay. In influx assays, choroid plexus was subsequently rinsed in ice-cold isotope-free HEPES-aCSF containing 2 mM ouabain, 20 µM bumetanide, and 1 mM BaCl_2_ (to prevent efflux of intracellular [^86^Rb^+^] during the washing procedure), followed by transfer to scintillation vials containing 100 µl Solvable (6NE9100, Perkin Elmer) to dissolve the choroid plexus. For efflux, the choroid plexus was swiftly rinsed in 37 °C isotope-free HEPES-aCSF, then transferred into new wells containing 37 °C isotope-free HEPES-aCSF with or without 20 µM bumetanide, at 10 s intervals. For each time point, 200 µl of the surrounding HEPES-aCSF was collected into a scintillation vial. At the end of the experiment, the choroid plexus was placed into a scintillation vial containing 200 µl Solvable to dissolve the choroid plexus. Isotope content was determined in 2 ml Ultima Gold^™^ XR scintillation liquid (6013119, Perkin Elmer) using the Tri-Carb 2900TR Liquid Scintillation Analyzer (Packard). The ^86^Rb^+^ activity was corrected for extracellular background using ^3^H-mannitol [57, 69]. Efflux data are shown as the natural logarithm of the ^86^Rb^+^ activity at each time point (A_T_) normalized to the initial ^86^Rb^+^ activity (A_0_) as a function of time. The slope from linear regression analysis was used to determine the ^86^Rb^+^ efflux rate constant [57, 69].

### Statistical analysis

Data analysis and statistical tests were carried out using Graphpad Prism version 9 (GraphPad software). All data were tested for normality of distribution using Shapiro–Wilk test prior to statistical analysis with Student's unpaired t-test, one-way ANOVA with Sidaks multiple comparisons test, and simple linear regression, as stated in figure legends. Data are displayed as the mean and standard error of the mean (SEM) with a p-value < 0.05 employed to define statistical significance. Outliers were determined with Grubbs’ test, when indicated in figure legend.

## Results

### HFD causes elevated ICP in rodents

**Fig. 1:**
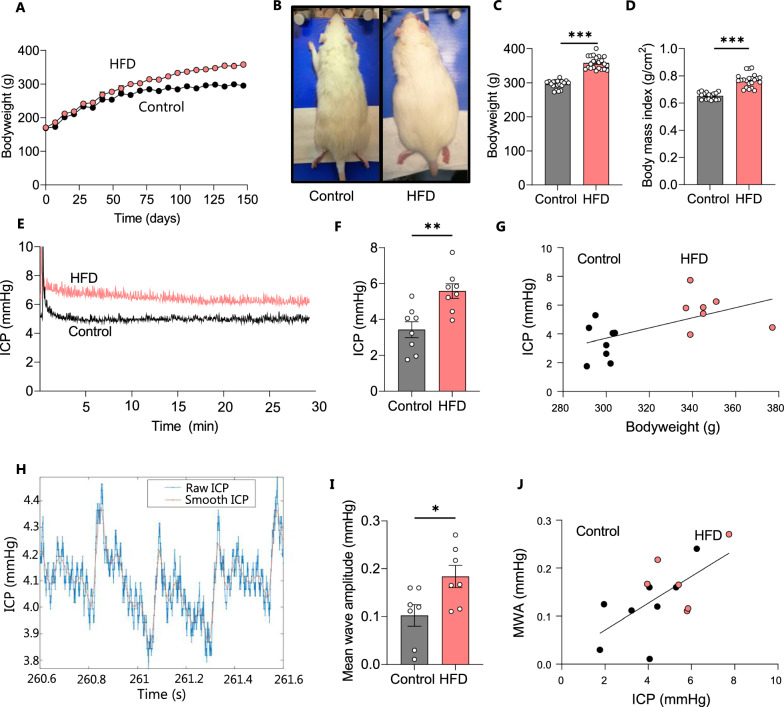
HFD leads to increased bodyweight and intracranial pressure in female rats.** A** Bodyweight increase as a function of time of all control (n = 14) and HFD-fed (n = 17) rats included in the study with the visibly larger rats illustrated in **B**, the bodyweight at the time of experiments illustrated in **C,** and the BMI at the time of experiment illustrated in **D. E** Representative ICP traces of control and HFD-fed rats with the last 15 min employed for the quantification, summarized in **F**. **G** ICP correlates with bodyweight across control and HFD-fed rats (R^2^ = 0.34, p < 0.05, n = 16). **H** Representative ICP trace with “raw” (blue line) and “smoothed” (red line) signals, the latter used to calculate the mean wave amplitude (MWA), represented in **I** (n = 7 af each). **J** Correlation analysis of MWA as a function of the ICP, n = 14, R^2^ = 0.40, p < 0.05. Statistical significance evaluated with Student’s unpaired t-test and results shown as mean ± SEM. *p < 0.05, **p < 0.01, ***p < 0.001

Obesity in humans is commonly attributed to myriad factors including genetic predisposition, macro- and micro-environmental factors, and lack of physical activity, but the primary recognized feature is consumption of energy rich diet [70]. In order to recapitulate this primary condition in a rodent model, female Wistar rats, aged 6 weeks, were divided into two groups, high fat diet-fed (HFD) and control, and were respectively fed either a HFD (60% Kcal from fat) or a nutrient source-matched control diet (10% Kcal from fat) for 21 weeks. HFD-fed rats gained weight at an accelerated rate (Fig. 1A) and were visibly larger (Fig. 1B) and approximately 21% heavier than their control counterparts on the day of experimentation (HFD: 358 ± 4 g, n = 22 vs control: 296 ± 3 g, n = 19, p < 0.001, Fig. 1C), with elevated BMI (HFD: 0.77 ± 0.01 g/cm^2^, n = 22 vs control: 0.66 ± 0.01 g/cm^2^ n = 19, p < 0.001, Fig. 1D). To determine if the HFD rats displayed the elevated ICP characteristic of the IIH patients, the ICP was measured with an epidural pressure probe in the anesthetized rats (Fig. 1E). The ICP was ~ 65% elevated in the HFD-fed rats compared to the control rats (HFD: 5.6 ± 1.2 mmHg, n = 8 vs control: 3.4 ± 1.3 mmHg, n = 8, p < 0.05, Fig. 1F), with the ICP displaying correlation to the body mass across the two groups of experimental rats (n = 16, R^2^ = 0.34, p < 0.05, Fig. 1G). These data demonstrate that dietary-induced body mass elevation can cause the ICP elevation in rodents that is a hallmark feature of IIH in patients. IIH patients display altered pulsatile ICP as evidenced in abnormal ICP mean wave amplitude, possibly arising from IIH-related changes in the neurovasculature of these patients [47]. To determine if such ICP pattern disturbances were present in the HFD-fed rats, ICP waveform analysis quantified the amplitude change of the cardiac waveform (Fig. 1H), revealing elevated mean wave amplitude (MWA) in HFD rats (HFD: 0.18 ± 0.02 mmHg, n = 7 vs control: 0.10 ± 0.02, n = 7 p < 0.05, Fig. 1I). MWA correlates with ICP across the two groups of experimental rats (n = 14, R^2^ = 0.40, p < 0.05, Fig. 1J). We therefore employed this model to determine whether obesity-induced disturbances in CSF dynamics could underlie the elevated ICP in these animals.

### HFD-induced ICP elevation coincides with increased resistance to CSF drainage, but not increased CSF production

**Fig. 2:**
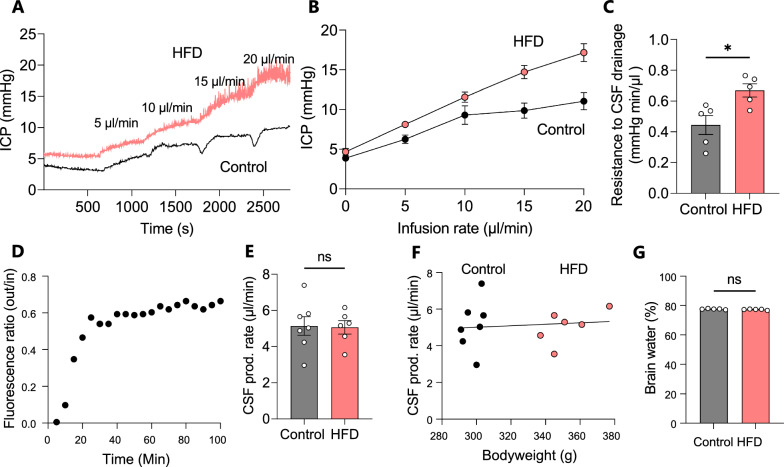
HFD increases resistance to CSF drainaige, but does not alter CSF production measured by ventriculo-cisternal perfusion.** A** Representative ICP traces of control and HFD-fed rats at successive CSF infusion rates, with averaged ICP values quantified in **B** and R_out_ quantified in **C** (n = 5 of each)**. D** Representative trace of fluorescent dye dilution over the course of a ventriculo-cisternal perfusion assay in a control rat with the final 30 min employed for quantification of the CSF production rate illustrated in **E** Rate of CSF production in control and HFD-fed rats, **F** no correlation was observed between bodyweight and CSF production rate. **G** The percentage brain water of HFD-fed and control rats, (n = 5 of each). Statistical significance evaluated with Student's unpaired t-test and simple linear regression and results shown as mean ± SEM. *p < 0.05, *ns*  not significant

To determine whether HFD-fed rats mimic the elevated CSF outflow resistance (R_out_) detected in most IIH patients [71] ICP was measured at varying CSF infusion rates as previously described [51], Fig. 2A, B. The R_out_ was significantly elevated in HFD-fed rats relative to controls (HFD: 0.67 ± 0.04 mmHg min/µl, n = 5 vs control: 0.44 ± 0.06, n = 5 p < 0.05, Fig. 2C), which suggests a reduced rate of CSF drainage.

To determine if HFD-mediated increased ICP arises subsequent to an increased CSF production rate, we assessed this parameter in vivo in anesthetized and mechanically ventilated rats with the ventriculo-cisternal perfusion technique [57]. Here, heated and gas-equilibrated aCSF containing a fluorescent dextran is delivered continuously into the lateral ventricle with concomitant fluid collection from a cisterna magna puncture. The dextran dilution taking place during the fluid passage through the ventricles originates from fluid secreted into the ventricles and can thus be employed to calculate the rate of CSF production (Fig. 2D). No significant difference in the rate of CSF production was observed between control and HFD-fed rats (HFD: 5.06 ± 0.91 µl/min, n = 7 vs control: 5.14 ± 1.38 µl/min, n = 6, p = 0.91, Fig. 2E) with no correlation between bodyweight and CSF production rate (n = 13, R^2^ = 0.01, p = 0.73, Fig. 2F). These data indicate that the elevated body weight elicited by HFD is not sufficient to significantly increase CSF production. CSF production was also assessed using the LI-COR technique, which relies on injection of a fluorescent dye into the lateral ventricle of the anaesthetized rat, after which the rat is swiftly transferred to the whole animal fluorescent reader and the fluorescence dispersion rate obtained as a proxy for the CSF secretion rate [46, 49, 57, 72]. This technique also revealed no significant elevation of CSF production in HFD -fed rats relative to control rats (Additional file 1). To determine whether the observed increase in ICP associated with excess brain water, the total brain water content was assessed. The brain water percentage was not significantly different between the two groups (HFD: 77.6 ± 0.3%, n = 5 vs control: 77.6 ± 0.3%, n = 5, p = 0.16, Fig. 2G). The increase in ICP observed in HFD-fed rats therefore does not appear to arise solely from an increased brain water content.

### The HFD-mediated ICP elevation is not reflected in choroid plexus gene transcription

**Fig. 3:**
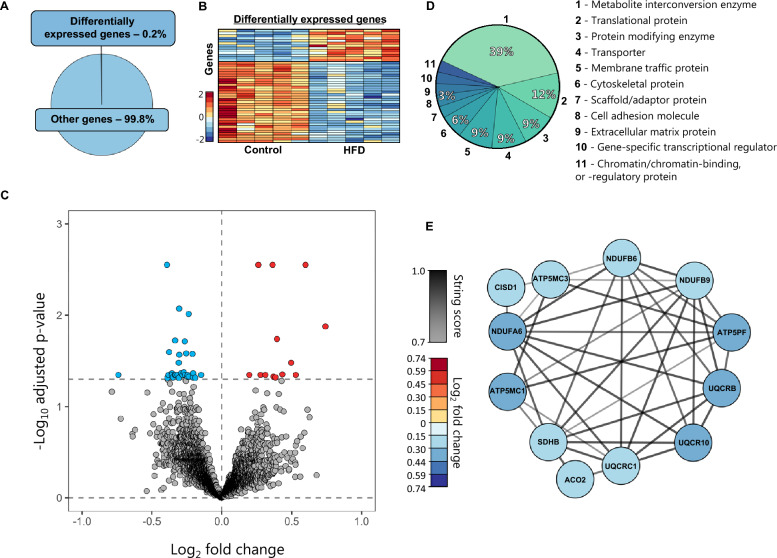
Negligible transcriptomic changes in choroid plexus of HFD-fed rats. **A** illustrates a pie chart of the percentage differentially expressed genes. **B** A heatmap of the differentially expressed genes in the individual rats, coloured based on the Z-score of normalized fold changes (n = 5 of each). **C** Volcano plot of all transcripts detected in the choroid plexus of control and HFD-fed rats with differentially expressed genes (Adjusted p-value < 0.05) marked in red (upregulated) or blue (downregulated). **D** GO enrichment analysis of the protein classes. **E** Association protein network analysis of the highest order cluster of genes. The protein nodes are coloured based on log_2_FC (downregulated in blue) and the connecting lines coloured based on the string confidence score from 0.7 (grey) to 1.0 (black)

The choroid plexus is the master controller of brain fluid secretion in the mammalian brain [42] and HFD-dependent modulation of its cellular and molecular components could potentially influence brain water dynamics in various ways. To obtain an unbiased manner of revealing potential HFD-mediated changes in functional properties of the choroid plexus, we performed RNA sequencing (RNAseq) of excised choroid plexus obtained from rats fed control diet or HFD. Of the 21,401 expressed genes detected in the choroid plexus (Additional file 2), only 46 of these (0.2%) were differentially expressed between the two groups (Fig. 3A and Additional file 3). A heatmap of the gene expression properties demonstrated that the majority of the differentially expressed genes were downregulated in HFD-fed rats compared to rats receiving control diet (Fig. 3B). A volcano plot demonstrated that of the 46 differentially expressed genes, 33 of these (72%) were downregulated and 13 of these (28%) upregulated (Fig. 3C and Additional file 3). To reveal the functional categories of the differentially expressed genes, we employed GO enrichment analysis (see Methods) to classify 33 of these into protein classes, revealing differentially expressed transcripts categorized as: metabolite interconversion enzymes (39%, 13/33), translational proteins (12%, 4/33), protein-modifying enzymes (9%, 3/33), membrane traffic proteins (9%, 3/33), and transporters (9%, 3/33)—with the remaining 21% scattered over various categories (Fig. 3D). The three differentially expressed transport proteins, ATP5MC1, ATP5MC3, and ATP5PF, though associated with a larger network of differentially expressed genes in the HFD-fed rat choroid plexus (Fig. 3E), are mitochondrial transporters of ATP complex V, not anticipated to be directly associated with CSF formation across the choroid plexus plasma membrane. Overall, the transcript profile of choroid plexus obtained from HFD-fed rats provides no direct indication of a choroid plexipathology as a contributor to the elevated ICP detected in the HFD-fed rats.

### CSF androgens as a contributing factor to IIH-related CSF dynamics

**Fig. 4:**
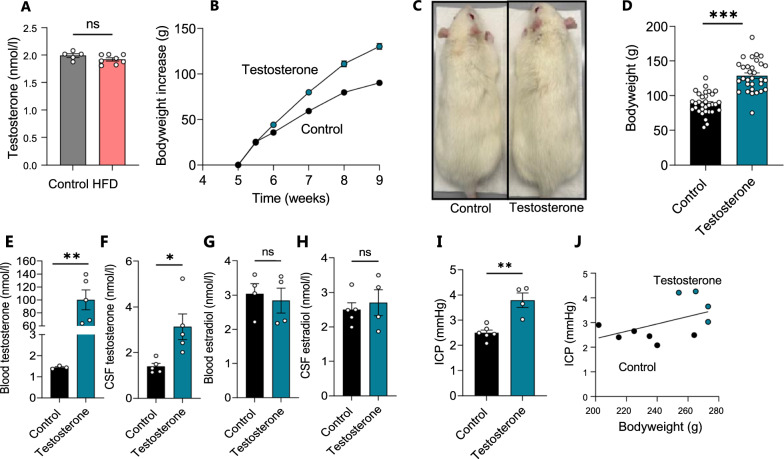
Testosterone treatment increases bodyweight, testosterone levels, and ICP**. A** CSF levels of testosterone quantified with LC–MS (n = 5–7). **B** Bodyweight increase as a function of time in all rats treated with testosterone (n = 25) or vehicle (n = 26), with **C** illustrating a visibly larger testosterone-treated rat and **D** their bodyweight at the time of experiments. **E-F** Plasma (**E,** n = 3–5) and CSF (**F,** n = 5) levels of testosterone quantified with LC–MS (after one outlier removed from the control group in **E**). **G**–**H** Plasma (**G,** n = 4) and CSF (**H,** n = 4–5) levels of 17β-estradiol quantified with LC–MS (after one outlier removed from the testosterone group in **G** and** H**).** I** quantification of ICP in control and testosterone-treated rats (n = 4–6). **J** Correlation analysis of ICP with bodyweight in control and testosterone-treated rats (R^2^ = 0.23, p = 0.16, n = 10). Statistical significance evaluated with Student’s unpaired t-test and results shown as mean ± SEM. *p < 0.05, **p < 0.01, ***p < 0.001, *ns* not significant

To determine whether CSF extracted from the HFD-fed rats differed in testosterone levels compared to their lean counterparts, as observed for IIH patients [39, 41], we performed liquid chromatography-mass spectrometry (LC–MS) analysis on CSF extracted from HFD-fed and control rats. HFD did not induce an elevated testosterone level in the rat CSF (HFD: 1.92 ± 0.03 nmol/l, n = 8 vs control: 1.99 ± 0.04 nmol/l, n = 5, p = 0.17, Fig. 4A), indicating that the HFD-mediated 20% increase in bodyweight alone was not sufficient to cause the hormonal disturbances characteristic of female IIH patients. With the HFD-fed rats not becoming obese, but merely overweight, they may not reach the point at which testosterone dysregulation occurs, and/or such phenomenon may not be linked to bodyweight in the rodent model. Therefore, to determine if elevated CSF testosterone, in itself, could directly modulate CSF dynamics, we subjected lean female Wistar rats to a four-week testosterone treatment regimen [54]. Prior to the initiation of the testosterone regimen, the average weight of the test rats was 146 ± 1.2 g, n = 63, but the weight of the testosterone-treated (TT) rats increased faster than that of the control rats (Fig. 4B). At the time of experiments, the testosterone-treated rats were visibly larger (Fig. 4C) and significantly heavier than their control counterparts (TT: 129 ± 4.1 g, n = 31 vs control: 88.4 ± 2.8 g, n = 32, p < 0.001, Fig. 4D). Quantification of plasma and CSF levels of testosterone were performed with LC–MS/MS and revealed elevated testosterone levels in the plasma samples (TT: 100 ± 34 nmol/l, n = 5 vs control: 1.4 ± 0.1 nmol/l, n = 3, p < 0.01, Fig. 4E), but more importantly; also elevated in the CSF (TT: 3.13 ± 1.26 nmol/l, n = 5 vs control: 1.39 ± 0.29 nmol/l, n = 5, p < 0.05, Fig. 4F). The 17β-estradiol level, on the other hand, was not significantly different between the two groups in either the blood (TT: 2.84 ± 0.72 nmol/l, n = 4 vs control: 3.04 ± 0.59 nmol/l, n = 4, p = 0.69, Fig. 4G) or the CSF (TT: 2.70 ± 0.75 nmol/l, n = 4 vs control: 3.04 ± 0.59 nmol/l, n = 5, p = 0.51, Fig. 4H). Of the panel of other steroid hormones and precursors tested during the mass spectrometry analysis, none was significantly elevated in the testosterone-treated rats (Additional file 4). ICP measurement in control and testosterone-treated rats revealed significantly elevated ICP in testosterone-treated rats relative to controls (TT: 3.79 ± 0.29 mmHg, n = 4 vs control: 2.50 ± 11 mmHg, n = 6, p < 0.01, Fig. 4E). To determine whether the testosterone-induced bodyweight increase could contribute to elevated ICP, we performed correlation analysis of the ICP versus the rat bodyweight. With the observed lack of correlation between these two parameters (n = 10, R^2^ = 0.234, p = 0.156, Fig. 4F), the obtained results suggest a testosterone-induced elevation of ICP independent of the rat bodyweight.

**Fig. 5:**
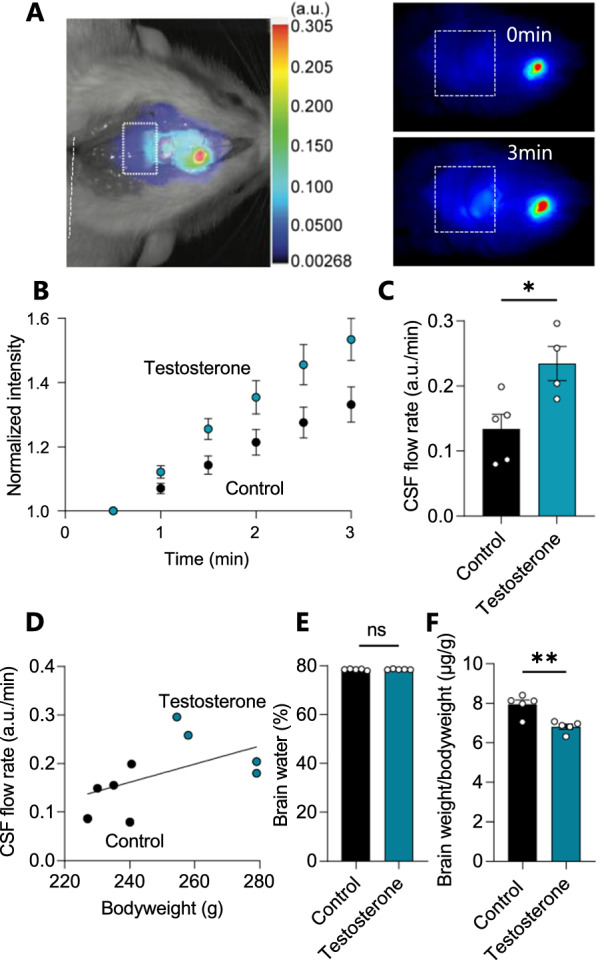
CSF flow increases with testosterone treatment. **A** Representative image of a rat after injection of IRDye 800CW carboxylate dye (superimposed pseudo-color). The square placed in line with lambda indicates the area of dye content quantification, as illustrated in the representative images obtained at t = 0 min and t = 3 min in control rats. **B** The dye intensity normalized to that obtained in the first image and plotted as a function of time representing flow rate, n = 4–5. **C** Quantification of the dye intensity (flow rate) determined from linear regression in **B** over the 3 min time window (with one outlier removed from the testosterone group) in arbitrary units (a.u.). **D** The CSF flow rate as a function of bodyweight did not display significant correlation with bodyweight in control and testosterone-treated rats (n = 9, R^2^ = 0.27, p = 0.16). **E** Percentage brain water in control and testosterone-treated rats (n = 5 of each). **F** The brain weight relative to the bodyweight of control and testosterone-treated rats (n = 5 of each). Statistical significance evaluated with Student’s unpaired t-test and results shown as mean ± SEM. *p < 0.05, ** p < 0.01, ns = not significant

To determine if the elevated testosterone modulated the rate of CSF secretion, the LI-COR imaging system was used to acutely assess the CSF flow in testosterone-treated female Wistar rats (Fig. 5A). The testosterone-treated animals displayed a near doubling of the dye flow (TT: 0.24 ± 0.05 a.u./min, n = 4 vs control: 0.13 ± 0.05 a.u./min, n = 5, p < 0.05, Fig. 5B, C) suggesting an elevated rate of CSF secretion. Naïve rats treated acutely with intracerebroventricular testosterone immediately preceding dye injection showed no effects on CSF flow (Additional file 5). To determine whether the testosterone-induced bodyweight increase could contribute to the elevated rate of CSF secretion, we performed correlation analysis of the CSF secretion rate versus the rat bodyweight. With the observed lack of correlation between these two parameters (n = 9, R^2^ = 0.27, p = 0.16, Fig. 5D), the obtained results suggest a testosterone-induced elevation of the CSF secretion rate independently of the rat bodyweight. Such testosterone-induced increase in the rate of CSF secretion could lead to brain water accumulation and thus contribute to the elevated ICP observed in the IIH patients. The brain water content of testosterone-treated rats was assessed with the dry–wet weight technique and revealed no significant difference in the brain water percentages between the two experimental groups (TT: 78.5 ± 0.2%, n = 5 vs control: 78.4 ± 0.3%, n = 5, p = 0.71, Fig. 5E) The brain weight relative to bodyweight was decreased in these animals (TT: 6.81 ± 0.13 µg/g bodyweight, n = 5 vs control: 7.94 ± 0.23 µg/g bodyweight, n = 5, p < 0.01, Fig. 5F), likely due to the excess bodyweight gain induced by testosterone treatment.

### Transport activity of NKCC1, not the Na^+^/K^+^-ATPase, is affected by testosterone treatment

**Fig. 6:**
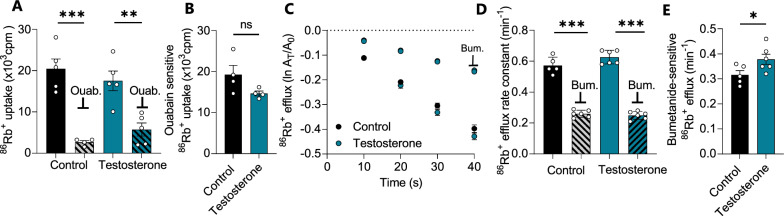
Choroid plexus NKCC1 activity is elevated testosterone-treated rats. **A** ^86^Rb^+^ influx into isolated choroid plexus from control and testosterone-treated rats in the absence and presence of ouabain (Ouab, 2 mM), n = 4–5 (after one outlier removed from the control-ouabain group). **B** The ouabain-sensitive (Na^+^/K^+^-ATPase-mediated) fraction of the ^86^Rb^+^ influx in choroid plexus from control and testosterone-treated rats (n = 4, after one outlier removed from the testosterone group). **C** Efflux of ^86^Rb^+^ from choroid plexus obtained from control rats or testosterone-treated rats in the absence or presence of NKCC1 inhibition by 20 µM bumetanide (BUM, n = 5–6 after one outlier removed from the control group). Y-axis is the natural logarithm of the amount left in the choroid plexus at time t (A_t_) divided by the amount at time 0 (A_0_). **D** The ^86^Rb^+^ efflux rate constant obtained with linear regression of the data from **C**. **E** The bumetanide-sensitive (NKCC1-mediated) fraction of the ^86^Rb^+^ efflux rate constant obtained from data in **D**. Results shown as mean ± SEM and statistical significance obtained with Student’s t-test (panels **B**, **E**) or ANOVA with Sidak's multiple comparisons test (panel **A**, **D**). *p < 0.05, **p < 0.01, ***p < 0.001, *ns* not significant

To resolve the molecular mechanisms underlying the testosterone-induced elevated CSF secretion, the activity of two choroid plexus transport mechanisms, the Na^+^/K^+^-ATPase and the Na^+^/K^+^/2Cl^−^ cotransporter (NKCC1), both known to contribute to CSF secretion [49] was assessed. To determine the contribution of the Na^+^/K^+^-ATPase to the increased CSF production observed in the testosterone-treated rats, its transport activity was determined in acutely excised choroid plexus from testosterone-treated rats and their control counterparts by influx assays with the radioisotope ^86^Rb^+^, as a congener of K^+^, in the absence and presence of the Na^+^/K^+^-ATPase inhibitor ouabain (Fig. 6A). The ouabain-sensitive fraction of the ^86^Rb^+^ uptake thus represents the Na^+^/K^+^-ATPase activity, which was not significantly different between the two experimental groups (TT: 14.7 ± 6.4 × 10^3^ cpm, n = 4 vs control: 19.2 ± 3.8 × 10^3^ cpm, n = 4, p = 0.25, Fig. 6B). Determination of NKCC1 activity was obtained with an efflux assay of pre-equilibrated ^86^Rb^+^ in the acutely excised choroid plexus in the absence and presence of the NKCC1 inhibitor bumetanide, Fig. 6C, D. The bumetanide-sensitive fraction of the ^86^Rb^+^ efflux thus represents the NKCC1 activity, which was significantly elevated in the choroid plexus obtained from the testosterone-treated rats (TT: 0.38 ± 0.05 min^−1^, n = 6 vs control: 0.32 ± 0.03 min^−1^, n = 5, p < 0.05, Fig. 6E). None of the transport parameters were altered by acute (1 h) exposure of the excised choroid plexus to testosterone (Additional file 5). The data suggest that Na^+^/K^+^-ATPase activation does not underlie the increased CSF flow observed after a four week testosterone treatment in female rats, but that testosterone-induced elevation of NKCC1 transport activity could contribute to the elevated CSF secretion rate observed in these rats.

## Discussion

Here, we demonstrate that mimicking of IIH patient characteristics (i.e., female sex, youth, and obesity) in a rat model, led to elevated ICP and R_out_, cardinal features of IIH. The elevated ICP in this rat cohortdid not occur with relevant functional changes in the CSF-secreting tissue or with altered CSF secretion rate. However, in rats treated with adjuvant testosterone, causing elevated levels of CSF testosterone (akin to that observed in IIH patients), we observed increased CSF secretion rates. We therefore speculate that CSF testosterone excess is a relevant contributing causal driver in IIH etiology.

Although IIH has been acknowledged as a recognized pathology for more than a century [73, 74], the underlying etiology remains unresolved. According to the Monro-Kellie doctrine, the volume of brain, blood, and CSF is constant. It follows that an increase in any of these compartments, in the absence of a balanced reduction of either of the others, leads to an elevated ICP. Elevated venous blood pressure has been proposed as the underlying pathology [18, 19], possibly originating from venous stenosis [28, 29], although the latter later questioned as stenosis does not seem to correlate with the clinical course of the disease [31, 75]. IIH-related stenosis may thus arise as a consequence of the elevated ICP rather than as its underlying cause [76–78], though there is ample evidence of alleviation of IIH symptoms by directly reducing venous pressure in patients with venous stenosis [36, 76]. The CSF volume in IIH patients may [20–22], or may not [79], be elevated and/or redistributed to the subarachnoid space [79] or the brain parenchyma, the latter promoting brain edema and/or slit ventricles [35, 80]. However, subsequent studies failed to detect diffuse brain edema [35, 81] or ventricular slits [79, 80, 82] as obligatory features of IIH patients. The outflow resistance is generally increased in IIH patients [24–26], although not necessarily [83]. With the uncertainty of IIH-related CSF flow disturbances leading to increased ICP and the invasive nature of some of these measurements, we employed a female rat model to seek to unravel the causative link between overweight and elevated ICP. The rats were fed a high fat diet to induce fat gain, as hyperphagia of calorie dense, high fat and palatable food is the principle manner in which the human patients become obese [84].

HFD-fed rats exhibited increased ICP as a function of their bodyweight, as previously demonstrated in a study using young female rats on a shorter duration obesogenic HFD program with similar weight gain, displaying similar increases in ICP, as well as indications of other IIH symptoms including reduced cerebrovascular compliance, papilledema, and headache [53] and aligned with earlier findings in a genetic rat obesity model deficient in the leptin receptor [85], thus manifesting one of the hallmark symptoms of IIH. In addition, the mean wave amplitude of the ICP dynamics was increased in HFD-fed rats and displayed correlation with the ICP elevation observed in these animals. This characteristic mimics the human condition and could indicate that the mean wave amplitude may increase with elevated ICP or, instead, be indicative of microvascular changes in the rat brain tissue similar to those observed in human IIH patients [30, 31, 33, 34]. HFD-fed rats presented with elevated R_out_, indicating reduced efficiency of CSF drainage similar to that observed in IIH patients [71]. Combined, the increased ICP, the reduced cerebrovascular compliance indicated by the elevated mean wave amplitude, and the elevated R_out_ observed in HFD-fed rats provide a compelling animal model for approximating several aspects of human IIH clinical presentation. Increased resistance to CSF drainage, as well as reduced compliance of the cerebrovascular system could be secondary to changes in venous pressure, which has been observed to increase in IIH patients [86], though these parameters were not measured in this rodent model. Previous studies in IIH patients revealed a correlation between CSF pressure, sagittal sinus pressure, and ICP [18], indicating the importance of considering the interplay between these parameters. The contribution of increased venous pressure in IIH remains to be determined; while it has been proposed that obesity-related increases in thoracic pressure can lead to increased venous pressure, these changes are not restricted to IIH patients [76, 87] and therefore may require other contributing factors, like those proposed here, to manifest in IIH symptoms. The observed ICP of the HFD- fed rats was approximately 65% higher than that of the control animals, which aligns with the earlier reports on such rat models [53, 85]. However, this ICP elevation is lower than the (minimum) doubling of the mean lumbar opening pressure observed in IIH patients compared to healthy control subjects [88]. Therefore, although these overweight rats appear to recapitulate the cardinal phenotype of IIH symptomatology, elevated ICP, this model cannot mimic the magnitude of ICP change seen in IIH. This discrepancy may, in part, be due to rodent obesity not being metabolically equivalent to that of human patients [84] and/or to the HFD-fed rats failing to reach the level of obesity (rat BMI roughly translated to a human BMI of 28, 17% increase over their lean counterparts) often observed in IIH patients (average BMI of 32 (range 20–70), 40% increase over their lean counterparts [89]). Similarly, while rat age and developmental cycle does not completely parallel that of humans, the 21 week HFD feeding program of these animals represents a shorter window (roughly equivalent to 12 human years), in which aspects of obesity, such as neuroendocrine imbalances and metabolic inflammation, can exert effects, relative to human IIH patients [90]. At 27 weeks old, rats have a roughly equivalent age of 19 human years, which would represent the early stages of IIH pathophysiology in human patients [90].

Elevated CSF secretion has been proposed to be associated with the elevated ICP in IIH patients [23, 27], although later questioned [83]. The elevated ICP in our HFD-fed rats did not associate with increased brain fluid content, or occur due to an increased rate of CSF secretion. Our findings contrast those of an earlier report in which rodent CSF secretion appeared increased following a high fat diet (but with no ICP measurements provided) [51]. Although we cannot explain this discrepancy, one possibility could be the degree and rapidity of weight gain, which has been shown to be an exacerbating factor in the presentation of IIH symptoms [89, 91]. In the previous study, female rats increased their weight 3.5-fold over a 7 week course, despite using a lower fat diet (45% vs. 60% in the current study) [51], whereas the female rats employed in the current study merely doubled their weight over a 21 week course, an important difference which may affect the appearance of IIH [91]. The small number of experimental animals (n = 3) employed in the previous study, the high volumes of experimentally infused aCSF, and/or the lack of mechanical ventilation of the animals during the prolonged anesthesia required for the procedure [51] may, in addition, contribute to the discrepancy between our findings. In support of the undisturbed CSF secretion observed in the HFD-fed rats, we observed negligible HFD-induced functional alterations in the CSF-secreting tissue choroid plexus, as determined from the transcriptomic profile of this tissue. Merely 46 of the > 21,000 transcribed genes obtained from the tissue (0.2%) were differentially expressed in the HFD-fed rat choroid plexus. Of these, only three were categorized as transport proteins, all localized to the mitochondria and all being downregulated, which, taken together, do not support an increase in choroid plexus-mediated CSF secretion in the rats on HFD. The network-related, downregulated genes in the HFD-fed rat choroid plexus encompass many components of the mitochondrial energetic machinery. HFD and obesity are known to promote inflammation and mitochondrial dysfunction in many brain areas, especially those with unique blood–brain barrier characteristics, like the hypothalamus, median eminence and the choroid plexus [92–94]. While obesity-related changes might be expected in this case, these mitochondrial disturbances are most likely to decrease CSF production [95], and may therefore not contribute directly to the IIH symptoms observed in these rats.

While female sex and obesity are two of the cardinal characteristics of most IIH patients, the great majority of obese females do not have IIH. Striking among biomarkers distinguishing IIH patients from unaffected obese females is elevation of testosterone [39, 41], which in all tested female IIH patients is increased to a level over double that of bodyweight-matched controls, but below that of male subjects. The HFD-fed rats did not mimic the elevated CSF testosterone levels, which may be due to their modest bodyweight gain and/or species difference in the response to overweight. Adjuvant testosterone treatment of lean female rats was then employed to mimic the elevated levels of testosterone in the IIH patients, resulting in greater increases in serum testosterone, but similar increases in CSF testosterone to those observed in IIH patients [39, 41]. The elevated CSF levels of testosterone caused an increased ICP and increased rate of CSF production in these rats, which were, at least in part, associated with augmented activity of the choroid plexus transport protein, NKCC1, which has been demonstrated as a key contributor to CSF secretion in mice, rats, and dogs [45, 49, 57, 96]. The testosterone-mediated effect on the choroid plexus transport protein, the CSF secretion rate, and the ICP only came about with prolonged treatment with testosterone (4 weeks) and was absent with acute (1 h) testosterone exposure of the excised choroid plexus. In contrast, a previous study with acute testosterone exposure of cultured rodent choroid plexus epithelial cells observed increased Na^+^/K^+^-ATPase activity [39], which we here failed to replicate in the ex vivo choroid plexus. Notably, the 17β-estradiol levels were unaffected in both HFD-fed rats and testosterone-treated rats, suggesting that the observed effects on ICP and CSF flow could not be accounted for by 17β-estradiol, as has been proposed as a possible contributor to IIH in patients [38]. However, the rats were not controlled for their oestrous cycle in this study, which may introduce cycle-dependent changes in choroid plexus that may mask testosterone-mediated changes in functionality and is thus considered a limitation to the study. NKCC1 hyperactivity has previously been demonstrated as a regulator of CSF production in another condition of disordered CSF dynamics, i.e., posthemorrhagic hydrocephalus [45–47], likely occurring with activation of the SPS1-related proline/alanine-rich kinase (SPAK) [45, 46], which is highly expressed in the choroid plexus [97]. The androgen receptor, a nuclear receptor targeted by testosterone [98], is also functionally expressed in the rodent choroid plexus [97, 99, 100]. Androgens induce expression of SPAK [101] and modulate NKCC1 activity [102] which, taken together with the well-established SPAK-dependent modulation of NKCC1 activity [45, 46, 103] provide a potential coupling between the elevated androgenic tone observed in IIH patients and elevated NKCC1-mediated CSF secretion rate. The observed increase in NKCC1 activity in testosterone-treated rats therefore offers a mechanistic explanation for the increased CSF flow observed in these rats. Elevated CSF testosterone in female IIH patients could thus lead to increased CSF production and potentially elevated ICP with prolonged exposure to this androgen, and thereby contribute to the etiology of the disease.

In conclusion, we here demonstrate that although HFD-fed rats gained weight and displayed elevated ICP, their weight gain and ICP increase did not fully reach levels observed in IIH patients. The ICP elevation could not be accounted for by CSF hypersecretion but, at least in part, by a decreased CSF drainage capacity (increased R_out_) that together recapitulate IIH symptoms and allow mechanistic insight into IIH etiology. When mimicking the elevated testosterone environment, as observed in patients with IIH, we observed elevated ICP and CSF hypersecretion in the experimental rats. Synergistic effects of obesity and hyperandrogenism may thus, if sustained for prolonged time and potentially associated with other IIH-promoting factors [30, 31, 88] provide a mechanistic coupling to the elevated ICP observed in the IIH patients. Future in vivo animal studies on aspects of the venous blood pressure and volume in addition to combining obesity and androgen excess may reveal further pieces of the IIH etiology, and androgen blockade, in the context of obesity, may elucidate avenues for therapeutic targeting in IIH.

## Supplementary Information

**Additional file 1: **HFD does not significantly increase CSF flow.

**Additional file 2: Table S2.**of all the genes transcribed in the choroid plexus of Wistar rats fed either control or high fat diet. Listed in the table is the Ensembl ID (Rnor_6.0) together with the Gene (gene symbol). The ‘‘Control diet’’ and ‘‘High fat diet’’ are the mean values, in transcript per million (TPM), of five wistar rats on either control diet or high fat diet. Together with the mean values arestandard error of the mean (SEM) of the five ‘‘Control diet’’ and ‘‘High fat diet’’, also in TPM. All values have been rounded to closest integer, and values under one are annotated as ‘‘< 1’’.

**Additional file 3: Table S3.**of all the differentially expressed genes in the choroid plexus of Wistar rats fed control diet or high fat diet. Differentially expressed genes are all genes with an adjusted P < 0.05. The table contains the Ensembl ID (Rnor_6.0) together with the Gene (gene symbol). The MeanBase (DEseq2) is the mean of the normalized transcript count values, divided by size factors, taken across all samples. Log2FC represents the log2 fold change from control diet to high fat diet. A positive Log2FC value thus indicates an upregulation of gene expression in the high fat diet fed rats. Adjusted P value is calculated by the Benjamini–Hochberg procedure (see methods).

**Additional file 4: **Mass spectrometry analysis of CSF and blood hormones.

**Additional file 5: **Testosterone treatment does not affect CSF flow or choroid plexus transport rate.

## Data Availability

The datasets used in the current study are available from the corresponding author on reasonable request. Raw RNAseq data are available at the NCBI GEO database with accession number GSE223582, https://www.ncbi.nlm.nih.gov/geo/query/acc.cgi?acc=GSE223582. Scripts and data analysis are available at: https://github.com/Sorennorge/-MacAulayLab-RNAseq3-Wistar

## References

[1] Mollan SP, Wakerley BR, Alimajstorovic Z, Mitchell J, Ottridge R, Yiangou A, Thaller M, Gupta A, Grech O, Lavery G, Brock K, Sinclair AJ (2021). Intracranial pressure directly predicts headache morbidity in idiopathic intracranial hypertension. J Headache Pain.

[2] Wakerley BR, Mollan SP, Sinclair AJ (2020). Idiopathic intracranial hypertension: update on diagnosis and management. Clin Med.

[3] Yri HM, Fagerlund B, Forchhammer HB, Jensen RH (2014). Cognitive function in idiopathic intracranial hypertension: a prospective case-control study. BMJ Open.

[4] Yri HM, Ronnback C, Wegener M, Hamann S, Jensen RH (2014). The course of headache in idiopathic intracranial hypertension: a 12-month prospective follow-up study. Eur J Neurol.

[5] Markey KA, Mollan SP, Jensen RH, Sinclair AJ (2016). Understanding idiopathic intracranial hypertension: mechanisms, management, and future directions. Lancet Neurol.

[6] Mollan SP, Davies B, Silver NC, Shaw S, Mallucci CL, Wakerley BR, Krishnan A, Chavda SV, Ramalingam S, Edwards J, Hemmings K, Williamson M, Burdon MA, Hassan-Smith G, Digre K, Liu GT, Jensen RH, Sinclair AJ (2018). Idiopathic intracranial hypertension: consensus guidelines on management. J Neurol Neurosurg Psychiatry.

[7] Mollan SP, Aguiar M, Evison F, Frew E, Sinclair AJ (2019). The expanding burden of idiopathic intracranial hypertension. Eye.

[8] Hornby C, Mollan SP, Botfield H, O'Reilly MW, Sinclair AJ (2018). Metabolic concepts in idiopathic intracranial hypertension and their potential for therapeutic intervention. J Neuroophthalmol.

[9] Adderley NJ, Subramanian A, Nirantharakumar K, Yiangou A, Gokhale KM, Mollan SP, Sinclair AJ (2019). Association between idiopathic intracranial hypertension and risk of cardiovascular diseases in women in the United Kingdom. JAMA Neurol.

[10] Mollan SP, Mytton J, Tsermoulas G, Sinclair AJ (2021). Idiopathic intracranial hypertension: evaluation of admissions and emergency readmissions through the hospital episode statistic dataset between 2002–2020. Life.

[11] Friesner D, Rosenman R, Lobb BM, Tanne E (2011). Idiopathic intracranial hypertension in the USA: the role of obesity in establishing prevalence and healthcare costs. Obes Rev.

[12] Mollan SP, Ali F, Hassan-Smith G, Botfield H, Friedman DI, Sinclair AJ (2016). Evolving evidence in adult idiopathic intracranial hypertension: pathophysiology and management. J Neurol Neurosurg Psychiatry.

[13] Andrews LE, Liu GT, Ko MW (2014). Idiopathic intracranial hypertension and obesity. Horm Res Paediatr.

[14] Yri HM, Wegener M, Sander B, Jensen R (2012). Idiopathic intracranial hypertension is not benign: a long-term outcome study. J Neurol.

[15] Mulla Y, Markey KA, Woolley RL, Patel S, Mollan SP, Sinclair AJ (2015). Headache determines quality of life in idiopathic intracranial hypertension. J Headache Pain.

[16] Sinclair AJ, Burdon MA, Nightingale PG, Ball AK, Good P, Matthews TD, Jacks A, Lawden M, Clarke CE, Stewart PM, Walker EA, Tomlinson JW, Rauz S (2010). Low energy diet and intracranial pressure in women with idiopathic intracranial hypertension: prospective cohort study. BMJ.

[17] Mollan SP, Mitchell JL, Ottridge RS, Aguiar M, Yiangou A, Alimajstorovic Z, Cartwright DM, Grech O, Lavery GG, Westgate CSJ, Vijay V, Scotton W, Wakerley BR, Matthews TD, Ansons A, Hickman SJ, Benzimra J, Rick C, Singhal R, Tahrani AA, Brock K, Frew E, Sinclair AJ (2021). Effectiveness of bariatric surgery vs community weight management intervention for the treatment of idiopathic intracranial hypertension: a randomized clinical trial. JAMA Neurol.

[18] Lalou A-D, Czosnyka M, Czosnyka ZH, Krishnakumar D, Pickard JD, Higgins NJ (2020). Coupling of CSF and sagittal sinus pressure in adult patients with pseudotumour cerebri. Acta Neurochir.

[19] Liu KC, Starke RM, Durst CR, Wang TR, Ding D, Crowley RW, Newman SA (2017). Venous sinus stenting for reduction of intracranial pressure in IIH: a prospective pilot study. J Neurosurg.

[20] Sorensen PS, Thomsen C, Gjerris F, Henriksen O (1990). Brain water accumulation in pseudotumour cerebri demonstrated by MR-imaging of brain water self-diffusion. Acta Neurochir Suppl.

[21] Raichle ME, Grubb RL, Phelps ME, Gado MH, Caronna JJ (1978). Cerebral hemodynamics and metabolism in pseudotumor cerebri. Ann Neurol.

[22] Moser FG, Hilal SK, Abrams G, Bello JA, Schipper H, Silver AJ (1988). MR imaging of pseudotumor cerebri. AJR Am J Roentgenol.

[23] Johanson CE, Duncan JA, Klinge PM, Brinker T, Stopa EG, Silverberg GD (2008). Multiplicity of cerebrospinal fluid functions: new challenges in health and disease. Cerebrospinal Fluid Res.

[24] Gideon P, Sorensen PS, Thomsen C, Stahlberg F, Gjerris F, Henriksen O (1994). Assessment of CSF dynamics and venous flow in the superior sagittal sinus by MRI in idiopathic intracranial hypertension: a preliminary study. Neuroradiology.

[25] Børgesen SE, Gjerris F (1987). Relationships between intracranial pressure, ventricular size, and resistance to CSF outflow. J Neurosurg.

[26] Bercaw BL, Greer M (1970). Transport of intrathecal 131-I risa in benign intracranial hypertension. Neurology.

[27] Belal T, Al Tantawy A-E, Sherif FM, Ramadan A (2020). Evaluation of cerebrospinal fluid flow dynamic changes in patients with idiopathic intracranial hypertension using phase contrast cine MR imaging. Egypt J Neurol Psychiatry Neurosurg.

[28] Teleb MS, Cziep ME, Lazzaro MA, Gheith A, Asif K, Remler B, Zaidat OO (2013). Idiopathic intracranial hypertension a systematic analysis of transverse sinus stenting. Interv Neurol.

[29] Farb RI, Vanek I, Scott JN, Mikulis DJ, Willinsky RA, Tomlinson G, terBrugge KG (2003). Idiopathic intracranial hypertension: the prevalence and morphology of sinovenous stenosis. Neurology.

[30] Eidsvaag VA, Hansson HA, Heuser K, Nagelhus EA, Eide PK (2018). Cerebral microvascular abnormalities in patients with idiopathic intracranial hypertension. Brain Res.

[31] Eide PK, Hansson HA (2022). A new perspective on the pathophysiology of idiopathic intracranial hypertension: role of the glia-neuro-vascular interface. Front Mol Neurosci.

[32] Eide PK, Hasan-Olive MM, Hansson HA, Enger R (2021). Increased occurrence of pathological mitochondria in astrocytic perivascular endfoot processes and neurons of idiopathic intracranial hypertension. J Neurosci Res.

[33] Hasan-Olive MM, Hansson HA, Enger R, Nagelhus EA, Eide PK (2019). Blood-brain barrier dysfunction in idiopathic intracranial hypertension. J Neuropathol Exp Neurol.

[34] Eide PK (2021). Abnormal intracranial pulse pressure amplitude despite normalized static intracranial pressure in idiopathic intracranial hypertension refractory to conservative medical therapy. Life.

[35] Bastin ME, Sinha S, Farrall AJ, Wardlaw JM, Whittle IR (2003). Diffuse brain oedema in idiopathic intracranial hypertension: a quantitative magnetic resonance imaging study. J Neurol Neurosurg Psychiatry.

[36] Gurney SP, Ramalingam S, Thomas A, Sinclair AJ, Mollan SP (2020). Exploring the current management idiopathic intracranial hypertension, and understanding the role of dural venous sinus stenting. Eye Brain.

[37] Wall M (2010). Idiopathic intracranial hypertension. Neurol Clin.

[38] Markey KA, Uldall M, Botfield H, Cato LD, Miah MA, Hassan-Smith G, Jensen RH, Gonzalez AM, Sinclair AJ (2016). Idiopathic intracranial hypertension, hormones, and 11beta-hydroxysteroid dehydrogenases. J Pain Res.

[39] O'Reilly MW, Westgate CS, Hornby C, Botfield H, Taylor AE, Markey K, Mitchell JL, Scotton WJ, Mollan SP, Yiangou A, Jenkinson C, Gilligan LC, Sherlock M, Gibney J, Tomlinson JW, Lavery GG, Hodson DJ, Arlt W, Sinclair AJ (2019). A unique androgen excess signature in idiopathic intracranial hypertension is linked to cerebrospinal fluid dynamics. JCI Insight.

[40] Sinclair AJ, Ball AK, Burdon MA, Clarke CE, Stewart PM, Curnow SJ, Rauz S (2008). Exploring the pathogenesis of IIH: an inflammatory perspective. J Neuroimmunol.

[41] Abdelghaffar M, Hussein M, Abdelkareem SA, Elshebawy H (2022). Sex hormones, CSF and serum leptin in patients with idiopathic intracranial hypertension. The Egypt J Neurol Psychiatry Neurosurg.

[42] MacAulay N, Keep RF, Zeuthen T (2022). Cerebrospinal fluid production by the choroid plexus: a century of barrier research revisited. Fluids Barriers CNS.

[43] Mokri B (2001). The Monro-Kellie hypothesis: applications in CSF volume depletion. Neurology.

[44] Proulx ST (2021). Cerebrospinal fluid outflow: a review of the historical and contemporary evidence for arachnoid villi, perineural routes, and dural lymphatics. Cell Mol Life Sci.

[45] Karimy JK, Zhang J, Kurland DB, Theriault BC, Duran D, Stokum JA, Furey CG, Zhou X, Mansuri MS, Montejo J, Vera A, DiLuna ML, Delpire E, Alper SL, Gunel M, Gerzanich V, Medzhitov R, Simard JM, Kahle KT (2017). Inflammation-dependent cerebrospinal fluid hypersecretion by the choroid plexus epithelium in posthemorrhagic hydrocephalus. Nat Med.

[46] Toft-Bertelsen TL, Barbuskaite D, Heerfordt EK, Lolansen SD, Andreassen SN, Rostgaard N, Olsen MH, Norager NH, Capion T, Rath MF, Juhler M, MacAulay N (2022). Lysophosphatidic acid as a CSF lipid in posthemorrhagic hydrocephalus that drives CSF accumulation via TRPV4-induced hyperactivation of NKCC1. Fluids Barriers CNS.

[47] Lolansen SD, Rostgaard N, Barbuskaite D, Capion T, Olsen MH, Norager NH, Vilhardt F, Andreassen SN, Toft-Bertelsen TL, Ye F, Juhler M, Keep RF, MacAulay N (2022). Posthemorrhagic hydrocephalus associates with elevated inflammation and CSF hypersecretion via activation of choroidal transporters. Fluids Barriers CNS.

[48] Karimy JK, Reeves BC, Damisah E, Duy PQ, Antwi P, David W, Wang K, Schiff SJ, Limbrick DD, Alper SL, Warf BC, Nedergaard M, Simard JM, Kahle KT (2020). Inflammation in acquired hydrocephalus: pathogenic mechanisms and therapeutic targets. Nat Rev Neurol.

[49] Oernbo EK, Steffensen AB, Razzaghi Khamesi P, Toft-Bertelsen TL, Barbuskaite D, Vilhardt F, Gerkau NJ, Tritsaris K, Simonsen AH, Lolansen SD, Andreassen SN, Hasselbalch SG, Zeuthen T, Rose CR, Kurtcuoglu V, MacAulay N (2022). Membrane transporters control cerebrospinal fluid formation independently of conventional osmosis to modulate intracranial pressure. Fluids Barriers CNS.

[50] Oernbo EK, Lykke K, Steffensen AB, Tollner K, Kruuse C, Rath MF, Loscher W, MacAulay N (2018). Cerebral influx of Na(+) and Cl(-) as the osmotherapy-mediated rebound response in rats. Fluids Barriers CNS.

[51] Alimajstorovic Z, Pascual-Baixauli E, Hawkes CA, Sharrack B, Loughlin AJ, Romero IA, Preston JE (2020). Cerebrospinal fluid dynamics modulation by diet and cytokines in rats. Fluids Barriers CNS.

[52] Alimajstorovic Z, Westgate CSJ, Jensen RH, Eftekhari S, Mitchell J, Vijay V, Seneviratne SY, Mollan SP, Sinclair AJ (2020). Guide to preclinical models used to study the pathophysiology of idiopathic intracranial hypertension. Eye.

[53] Westgate CSJ, Hagen SM, Israelsen IME, Hamann S, Jensen RH, Eftekhari S (2022). The impact of obesity-related raised intracranial pressure in rodents. Sci Rep.

[54] Yu WH (1989). Administration of testosterone attenuates neuronal loss following axotomy in the brain-stem motor nuclei of female rats. J Neurosci.

[55] Jones HC, Deane R, Bucknall RM (1987). Developmental changes in cerebrospinal fluid pressure and resistance to absorption in rats. Dev Brain Res.

[56] Heisey SR, Held D, Pappenheimer JR (1962). Bulk flow and diffusion in the cerebrospinal fluid system of the goat. Am J Physiol.

[57] Steffensen AB, Oernbo EK, Stoica A, Gerkau NJ, Barbuskaite D, Tritsaris K, Rose CR, MacAulay N (2018). Cotransporter-mediated water transport underlying cerebrospinal fluid formation. Nat Commun.

[58] Weisser JJ, Hansen CH, Poulsen R, Larsen LW, Cornett C, Styrishave B (2016). Two simple cleanup methods combined with LC-MS/MS for quantification of steroid hormones in in vivo and in vitro assays. Anal Bioanal Chem.

[59] Dobin A, Davis CA, Schlesinger F, Drenkow J, Zaleski C, Jha S, Batut P, Chaisson M, Gingeras TR (2013). STAR: ultrafast universal RNA-seq aligner. Bioinformatics.

[60] Li B, Dewey CN (2011). RSEM: accurate transcript quantification from RNA-Seq data with or without a reference genome. BMC Bioinform.

[61] Love MI, Huber W, Anders S (2014). Moderated estimation of fold change and dispersion for RNA-seq data with DESeq2. Genome Biol.

[62] Thissen D, Steinberg L, Kuang D (2002). Quick and easy implementation of the Benjamini-Hochberg procedure for controlling the false positive rate in multiple comparisons. J Edu Behav Stat.

[63] Love MI, Anders S, Kim V, Huber W (2015). RNA-Seq workflow: gene-level exploratory analysis and differential expression. F1000Res.

[64] Wickham H, Wickham H (2016). Data Analysis. ggplot2: elegant graphics for data analysis.

[65] Kolde R, Kolde M: Package ‘pheatmap’. R Package 1 (7). In Tartu: University of Tartu. Republic of Estonia. 2015.

[66] Mi H, Ebert D, Muruganujan A, Mills C, Albou LP, Mushayamaha T, Thomas PD (2021). PANTHER version 16: a revised family classification, tree-based classification tool, enhancer regions and extensive API. Nucleic Acids Res.

[67] Szklarczyk D, Gable AL, Nastou KC, Lyon D, Kirsch R, Pyysalo S, Doncheva NT, Legeay M, Fang T, Bork P, Jensen LJ, von Mering C (2021). The STRING database in 2021: customizable protein-protein networks, and functional characterization of user-uploaded gene/measurement sets. Nucleic Acids Res.

[68] Otasek D, Morris JH, Boucas J, Pico AR, Demchak B (2019). Cytoscape Automation: empowering workflow-based network analysis. Genome Biol.

[69] Keep RF, Xiang J, Betz AL (1994). Potassium cotransport at the rat choroid plexus. Am J Physiol.

[70] Ding D, Gebel K (2012). Built environment, physical activity, and obesity: what have we learned from reviewing the literature?. Health Place.

[71] Kaipainen AL, Martoma E, Puustinen T, Tervonen J, Jyrkkänen HK, Paterno JJ, Kotkansalo A, Rantala S, Vanhanen U, Leinonen V, Lehto SM, Iso-Mustajärvi M, Elomaa AP, Qvarlander S, Huuskonen TJ (2021). Cerebrospinal fluid dynamics in idiopathic intracranial hypertension: a literature review and validation of contemporary findings. Acta Neurochir.

[72] Barbuskaite D, Oernbo EK, Wardman JH, Toft-Bertelsen TL, Conti E, Andreassen SN, Gerkau NJ, Rose CR, MacAulay N (2022). Acetazolamide modulates intracranial pressure directly by its action on the cerebrospinal fluid secretion apparatus. Fluids Barriers CNS.

[73] Quincke H (1896). Ueber Meningitis serosa und verwandte Zustände. Dtsch Z Nervenheilkd.

[74] Nonne M (1904). Über Fälle vom Symptomenkomplex Tumor cerebri“ mit Ausgang in Heilung (Pseudotumor cerebri) Über letal verlaufene Fälle von „Pseudotumor cerebri“ mit Sektionsbefund. Dtsch Z Nervenheilkd.

[75] Riggeal BD, Bruce BB, Saindane AM, Ridha MA, Kelly LP, Newman NJ, Biousse V (2013). Clinical course of idiopathic intracranial hypertension with transverse sinus stenosis. Neurology.

[76] Friedman DI (2006). Cerebral venous pressure, intra-abdominal pressure, and dural venous sinus stenting in idiopathic intracranial hypertension. J Neuroophthalmol.

[77] King JO, Mitchell PJ, Thomson KR, Tress BM (2002). Manometry combined with cervical puncture in idiopathic intracranial hypertension. Neurology.

[78] Scoffings DJ, Pickard JD, Higgins JN (2007). Resolution of transverse sinus stenoses immediately after CSF withdrawal in idiopathic intracranial hypertension. J Neurol Neurosurg Psychiatry.

[79] Silbergleit R, Junck L, Gebarski SS, Hatfield MK (1989). Idiopathic intracranial hypertension (pseudotumor cerebri): MR imaging. Radiology.

[80] Barkatullah AF, Leishangthem L, Moss HE (2021). MRI findings as markers of idiopathic intracranial hypertension. Curr Opin Neurol.

[81] Wall M, Dollar JD, Sadun AA, Kardon R (1995). Idiopathic intracranial hypertension Lack of histologic evidence for cerebral edema. Arch Neurol.

[82] Jacobson DM, Karanjia PN, Olson KA, Warner JJ (1990). Computed tomography ventricular size has no predictive value in diagnosing pseudotumor cerebri. Neurology.

[83] Malm J, Kristensen B, Markgren P, Ekstedt J (1992). CSF hydrodynamics in idiopathic intracranial hypertension: a long-term study. Neurology.

[84] Wong SK, Chin KY, Suhaimi FH, Fairus A, Ima-Nirwana S (2016). Animal models of metabolic syndrome: a review. Nutr Metab.

[85] Uldall M, Bhatt DK, Kruuse C, Juhler M, Jansen-Olesen I, Jensen RH (2017). Choroid plexus aquaporin 1 and intracranial pressure are increased in obese rats: towards an idiopathic intracranial hypertension model?. Int J Obes.

[86] Sugerman HJ, DeMaria EJ, Felton WL, Nakatsuka M, Sismanis A (1997). Increased intra-abdominal pressure and cardiac filling pressures in obesity-associated pseudotumor cerebri. Neurology.

[87] Hannerz J, Ericson K (2009). The relationship between idiopathic intracranial hypertension and obesity. Headache.

[88] Thaller M, Homer V, Hyder Y, Yiangou A, Liczkowski A, Fong AW, Virdee J, Piccus R, Roque M, Mollan SP, Sinclair AJ (2022). The idiopathic intracranial hypertension prospective cohort study: evaluation of prognostic factors and outcomes. J Neurol.

[89] Daniels AB, Liu GT, Volpe NJ, Galetta SL, Moster ML, Newman NJ, Biousse V, Lee AG, Wall M, Kardon R, Acierno MD, Corbett JJ, Maguire MG, Balcer LJ (2007). Profiles of obesity, weight gain, and quality of life in idiopathic intracranial hypertension (Pseudotumor cerebri). Am J Ophthalmol.

[90] Sengupta P (2013). The laboratory rat: relating its age with human's. Int J Prev Med.

[91] Rowe FJ, Sarkies NJ (1999). The relationship between obesity and idiopathic intracranial hypertension. Int J Obes Relat Metab Disord.

[92] Langley MR, Yoon H, Kim HN, Choi C-I, Simon W, Kleppe L, Lanza IR, LeBrasseur NK, Matveyenko A, Scarisbrick IA. 2020 High fat diet consumption results in mitochondrial dysfunction oxidative stress and oligodendrocyte loss in the central nervous system. Biochimica et Biophysica Acta (BBA) Molecular Basis of Disease. 10.1016/j.bbadis.2019.165630 10.1016/j.bbadis.2019.165630 PMC7982965 31816440

[93] Lee CH, Suk K, Yu R, Kim MS (2020). Cellular contributors to hypothalamic inflammation in obesity. Mol Cells.

[94] Cavaliere G, Trinchese G, Penna E, Cimmino F, Pirozzi C, Lama A, Annunziata C, Catapano A, Mattace Raso G, Meli R, Monda M, Messina G, Zammit C, Crispino M, Mollica MP (2019). High-fat diet induces neuroinflammation and mitochondrial impairment in mice cerebral cortex and synaptic fraction. Front Cell Neurosci.

[95] Krzyzanowska A, Carro E (2012). Pathological alteration in the choroid plexus of Alzheimer's disease: implication for new therapy approaches. Front Pharmacol.

[96] Javaheri S, Wagner KR (1993). Bumetanide decreases canine cerebrospinal fluid production In vivo evidence for NaCl cotransport in the central nervous system. J Clin Invest.

[97] Andreassen SN, Toft-Bertelsen TL, Wardman JH, Villadsen R, MacAulay N (2022). Transcriptional profiling of transport mechanisms and regulatory pathways in rat choroid plexus. Fluids Barriers CNS.

[98] Davey RA, Grossmann M (2016). Androgen receptor structure, function and biology: from bench to bedside. Clin Biochem Rev.

[99] Santos CR, Duarte AC, Quintela T, Tomas J, Albuquerque T, Marques F, Palha JA, Goncalves I (2017). The choroid plexus as a sex hormone target: functional implications. Front Neuroendocrinol.

[100] Alves CH, Goncalves I, Socorro S, Baltazar G, Quintela T, Santos CR (2009). Androgen receptor is expressed in murine choroid plexus and downregulated by 5alpha-dihydrotestosterone in male and female mice. J Mol Neurosci.

[101] Qi H, Labrie Y, Grenier J, Fournier A, Fillion C, Labrie C (2001). Androgens induce expression of SPAK, a STE20/SPS1-related kinase, in LNCaP human prostate cancer cells. Mol Cell Endocrinol.

[102] Chinn GA, Sasaki Russell JM, Yabut NA, Maharjan D, Sall JW (2020). Androgenic modulation of the chloride transporter NKCC1 contributes to age-dependent isoflurane neurotoxicity in male rats. Anesthesiology.

[103] Alessi DR, Zhang J, Khanna A, Hochdorfer T, Shang Y, Kahle KT (2014). The WNK-SPAK/OSR1 pathway: master regulator of cation-chloride cotransporters. Sci Signal.

