# Supplementary material for: Modelling idiopathic intracranial hypertension in rats: contributions of high fat diet and testosterone to intracranial pressure and cerebrospinal fluid production
Source: Fluids Barriers CNS. 2023 Jun 16;20:44. doi: 10.1186/s12987-023-00436-1 (PMC10276479; doi:10.1186/s12987-023-00436-1)
Supplement: Supplementary file 1 — Additional file 1: HFD does not significantly increase CSF flow. [file 12987_2023_436_MOESM1_ESM.pptx]

## Slide 1
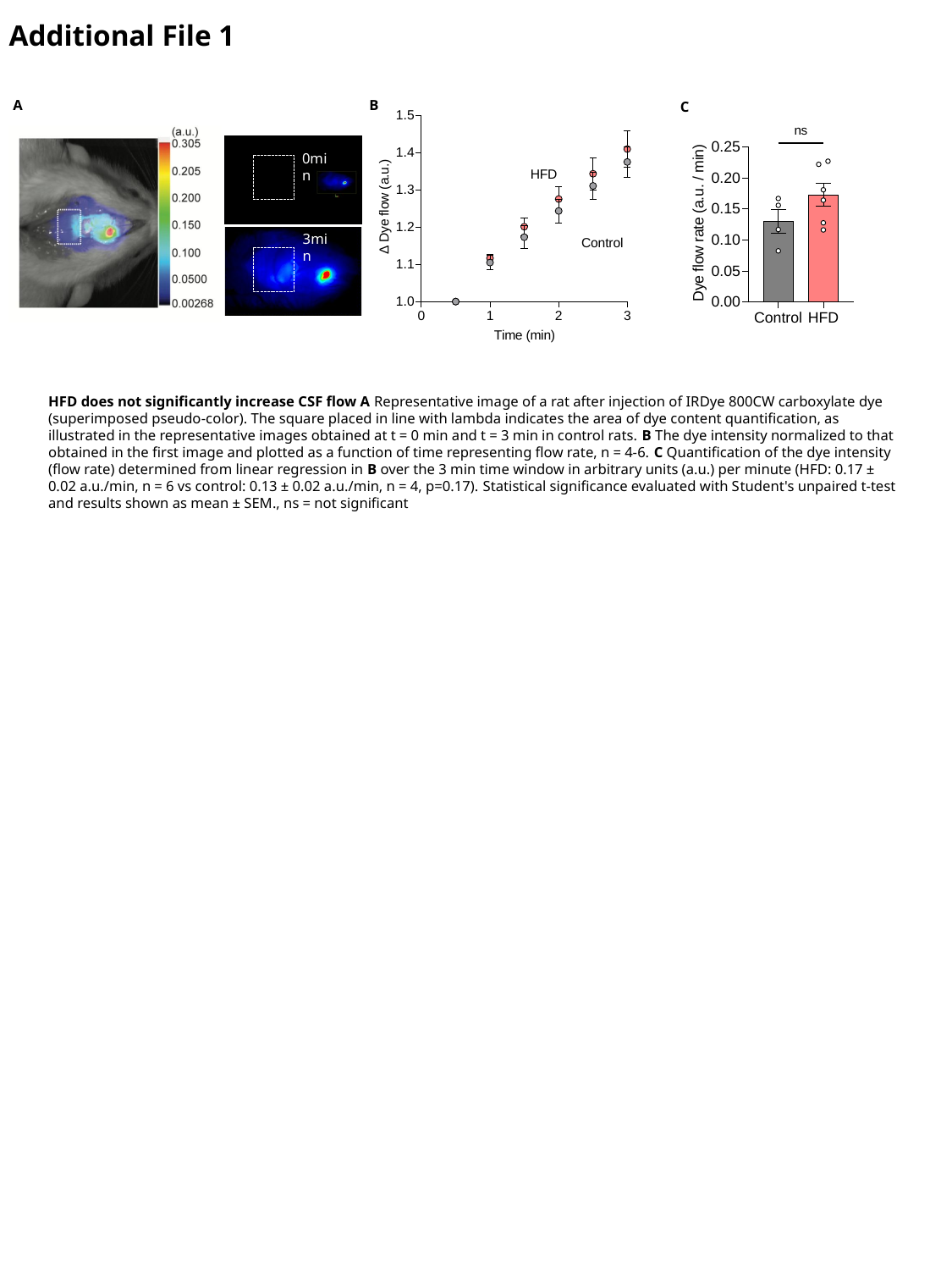

Additional File 1
A
B
C
0min
3min
HFD does not significantly increase CSF flow A Representative image of a rat after injection of IRDye 800CW carboxylate dye (superimposed pseudo-color). The square placed in line with lambda indicates the area of dye content quantification, as illustrated in the representative images obtained at t = 0 min and t = 3 min in control rats. B The dye intensity normalized to that obtained in the first image and plotted as a function of time representing flow rate, n = 4-6. C Quantification of the dye intensity (flow rate) determined from linear regression in B over the 3 min time window in arbitrary units (a.u.) per minute (HFD: 0.17 ± 0.02 a.u./min, n = 6 vs control: 0.13 ± 0.02 a.u./min, n = 4, p=0.17). Statistical significance evaluated with Student's unpaired t-test and results shown as mean ± SEM., ns = not significant
