# Supplementary material for: Modelling idiopathic intracranial hypertension in rats: contributions of high fat diet and testosterone to intracranial pressure and cerebrospinal fluid production
Source: Fluids Barriers CNS. 2023 Jun 16;20:44. doi: 10.1186/s12987-023-00436-1 (PMC10276479; doi:10.1186/s12987-023-00436-1)
Supplement: Supplementary file 4 — Additional file 4: Mass spectrometry analysis of CSF and blood hormones. [file 12987_2023_436_MOESM4_ESM.pptx]

## Slide 1
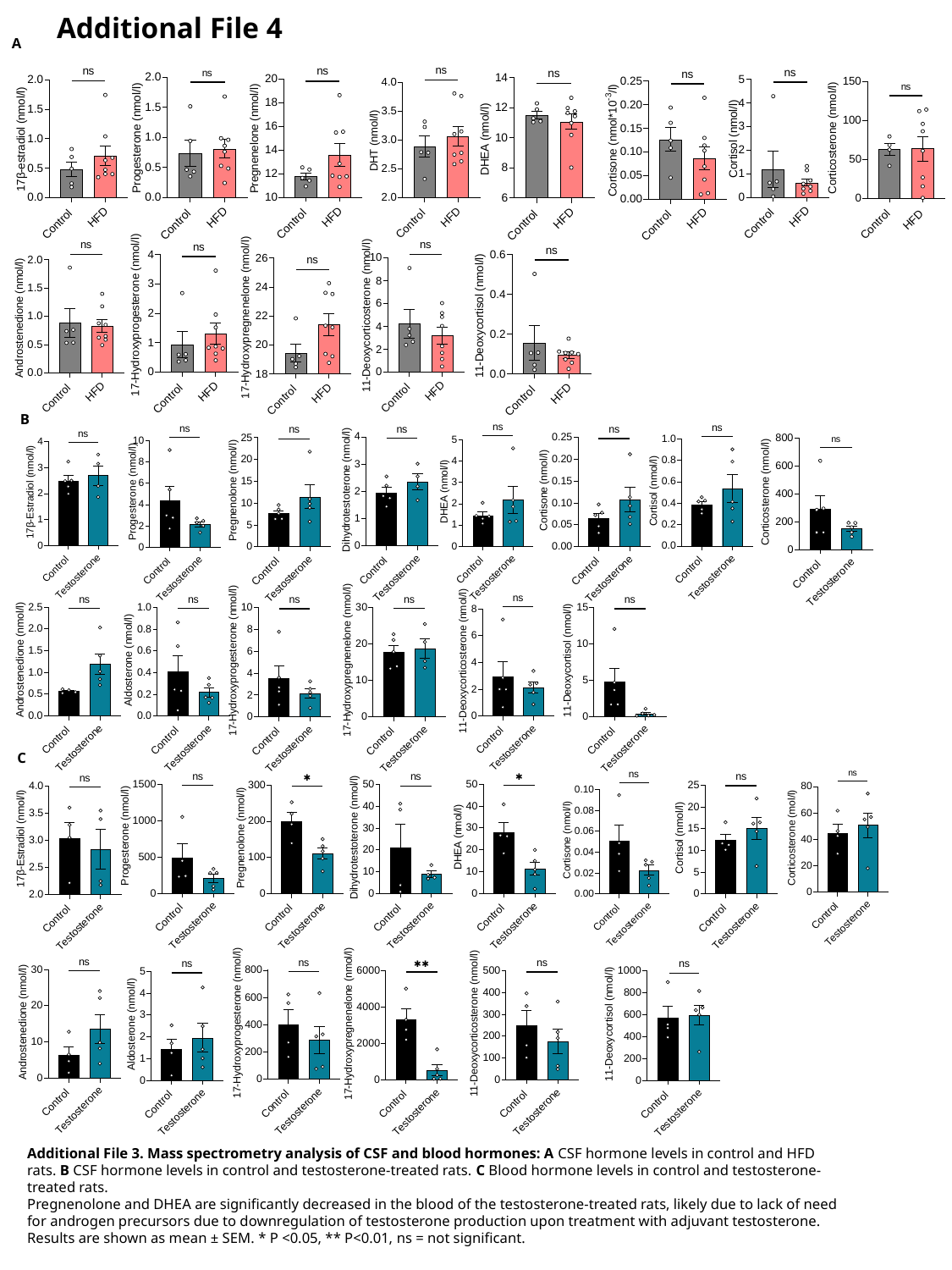

Additional File 4
A
B
C
Additional File 3. Mass spectrometry analysis of CSF and blood hormones: A CSF hormone levels in control and HFD rats. B CSF hormone levels in control and testosterone-treated rats. C Blood hormone levels in control and testosterone-treated rats.
Pregnenolone and DHEA are significantly decreased in the blood of the testosterone-treated rats, likely due to lack of need for androgen precursors due to downregulation of testosterone production upon treatment with adjuvant testosterone. Results are shown as mean ± SEM. * P <0.05, ** P<0.01, ns = not significant.
