# Supplementary material for: Modelling idiopathic intracranial hypertension in rats: contributions of high fat diet and testosterone to intracranial pressure and cerebrospinal fluid production
Source: Fluids Barriers CNS. 2023 Jun 16;20:44. doi: 10.1186/s12987-023-00436-1 (PMC10276479; doi:10.1186/s12987-023-00436-1)
Supplement: Supplementary file 5 — Additional file 5: Testosterone treatment does not affect CSF flow or choroid plexus transport rate. [file 12987_2023_436_MOESM5_ESM.pptx]

## Slide 1
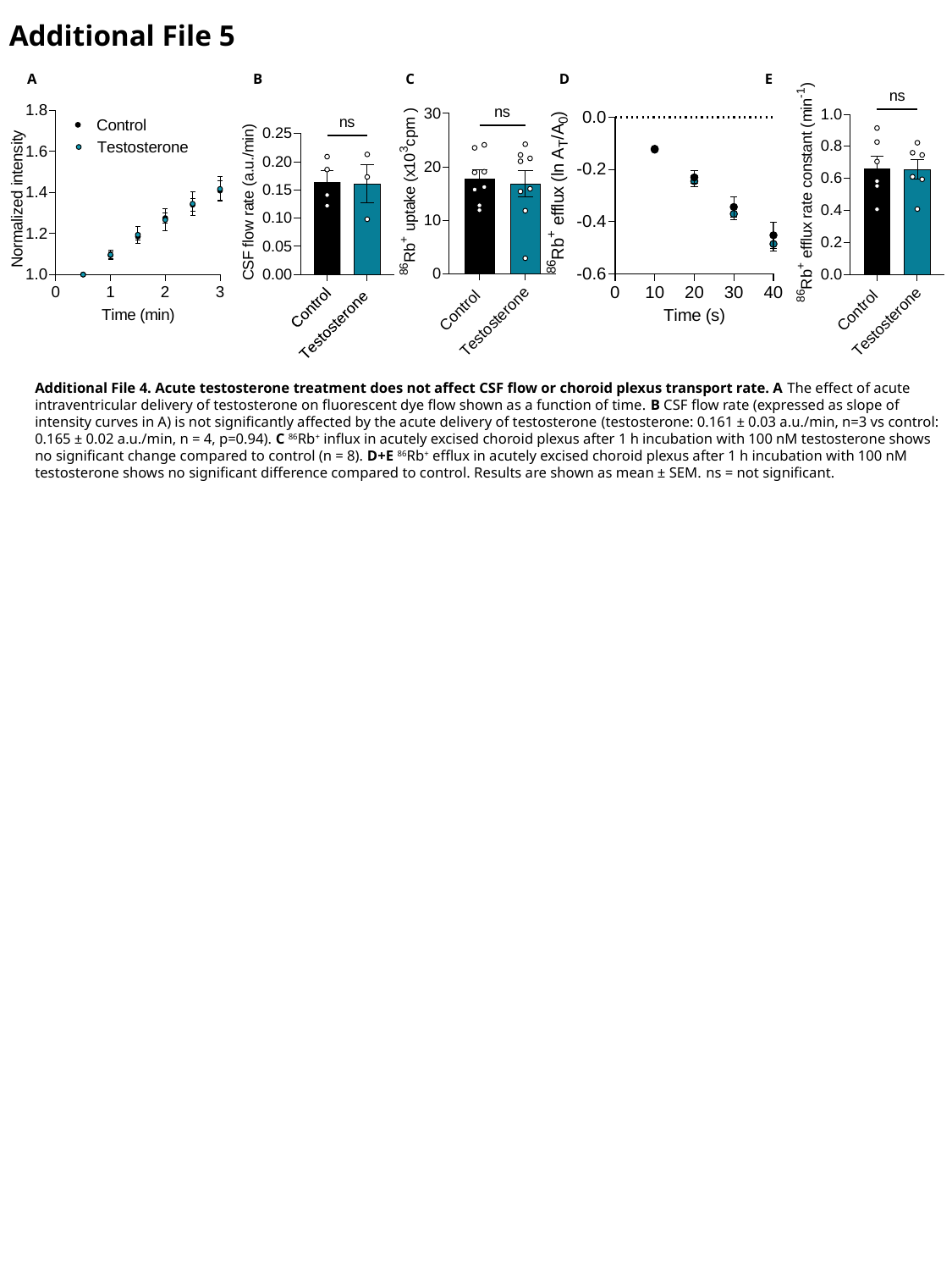

Additional File 5
C
D
E
A
B
Additional File 4. Acute testosterone treatment does not affect CSF flow or choroid plexus transport rate. A The effect of acute intraventricular delivery of testosterone on fluorescent dye flow shown as a function of time. B CSF flow rate (expressed as slope of intensity curves in A) is not significantly affected by the acute delivery of testosterone (testosterone: 0.161 ± 0.03 a.u./min, n=3 vs control: 0.165 ± 0.02 a.u./min, n = 4, p=0.94). C 86Rb+ influx in acutely excised choroid plexus after 1 h incubation with 100 nM testosterone shows no significant change compared to control (n = 8). D+E 86Rb+ efflux in acutely excised choroid plexus after 1 h incubation with 100 nM testosterone shows no significant difference compared to control. Results are shown as mean ± SEM. ns = not significant.
